# Hypokalemia-Induced Life-Threatening Arrhythmia in a Patient With Congestive Heart Failure

**DOI:** 10.7759/cureus.34971

**Published:** 2023-02-14

**Authors:** Hithem Fargaly, Rebecca J Schultz, Un Yong Chung, Noreen F Rossi

**Affiliations:** 1 Nephrology, Wayne State University Detroit Medical Center, Detroit, USA; 2 Nephrology, Wayne State University School of Medicine, Detroit, USA; 3 Nephrology, Wayne State University, Detroit, USA

**Keywords:** over-diuresis, acute kidney injury, congestive heart failure, medication reconciliation, ventricular tachycardia, hypokalemia

## Abstract

Ventricular tachyarrhythmias are common in patients with heart failure. It is one of the important preventable causes of death in these patient populations. Hypokalemia is prevalent in patients with heart failure due to various reasons. Hypokalemia can trigger ventricular arrhythmias through different mechanisms. In this case report, we present a middle-aged man with congestive heart failure (CHF) and an automated intracardiac defibrillator (AICD) on multiple diuretic medications (unintended) who presented with acute chest pain. He was found to have severe hypokalemia, hyponatremia, and an acute kidney injury. Interrogation of the AICD revealed multiple episodes of ventricular fibrillation. The patient was managed by holding his diuretic medications, cautious volume repletion, and potassium replacement. Fortunately, the patient showed rapid clinical improvement and his plasma potassium level improved. On discharge, we reconciled the patient’s medications to avoid the recurrence of hypokalemia from over-diuresis and arranged a close follow-up outpatient visit with his cardiologist.

## Introduction

Ventricular arrhythmias are common in patients with congestive heart failure (CHF) [1]. There are multiple postulated mechanisms that make patients with CHF more susceptible to ventricular arrhythmias. These mechanisms may be intrinsic to the heart or extrinsic. Important extrinsic causes include electrolyte disturbances, e.g., hypokalemia, hypomagnesemia, medication side effects, and substance use disorder, e.g., cocaine [2].

Hypokalemia is defined as a plasma potassium concentration of less than 3.5 mmol/dL. It may be categorized based on the plasma level as mild (3.0-3.5 mmol/dL), moderate (2.5-3.0 mmol/dL), and severe (<2.5 mmol/dL). It is a common electrolyte abnormality in both the inpatient and outpatient settings. In the inpatient setting, it may be seen in about 20% of hospitalized patients [3].

There is an increased incidence of hypokalemia in patients with cardiovascular disease: hypertension, CHF, and acute myocardial infarction.

Savarese et al found that 20.3% of patients with CHF experienced at least one episode of hypokalemia within one year of the study, and 3.7% experienced severe hypokalemia [4]. The most common etiology of hypokalemia in these patients is renal loss of potassium from diuretic therapy [5].

Hypokalemia has serious consequences including increased in-hospital mortality secondary to its effect on cardiac rhythm and blood pressure. Patients with CHF are significantly susceptible to sudden cardiac death from hypokalemia [6].

In this case report, we aim to demonstrate one of the life-threatening complications of hypokalemia in a patient with CHF. It also highlights the critical importance of medication reconciliation in preventing such complications.

## Case presentation

A 52-year-old man with a history of heart failure with reduced ejection fraction (HFrEF) secondary to alcoholic cardiomyopathy status post an Automatic Intracardiac Defibrillator (AICD) placement presented to our emergency department (ED) with left-sided chest pain.

Six days before the presentation, the patient had a sudden onset of chest pain that awakened him from sleep. He called his cardiologist who advised him to report to the ED in case of a recurrence of chest pain. Three days before the presentation, the chest pain recurred and the patient presented to our ED for evaluation. The chest pain was localized to the area of the chest over the AICD, it was non-radiating, sharp, intermittent, occurring generally in the mornings, lasting a few minutes with spontaneous resolution, and associated with shortness of breath. He did not recall any aggravating or alleviating factors for the chest pain. He denied a history of similar episodes of chest pain.

The patient endorsed nausea, decreased appetite, and frequent urination of a large amount of urine. He denied fever, cough, orthopnea, paroxysmal nocturnal dyspnea, palpitation, leg swelling, vomiting, diarrhea, dysuria, or hematuria. 

The patient had a past medical history of paroxysmal atrial fibrillation and chronic kidney disease (stage G2). His medication profile per electronic health record (EHR) included metolazone 2.5 mg daily, torsemide 40 mg daily, amiodarone 200 mg twice daily, apixaban 5 mg twice daily, aspirin 81 mg daily, atorvastatin 20 mg daily, carvedilol 6.25 mg twice daily, oral potassium 20 mmol daily, sacubitril-valsartan 97-103 mg twice daily, and spironolactone 25 mg daily. The patient brought in some of his medication bottles. We performed medication reconciliation and found that there was a discrepancy between the patient’s EHR medication profile and what the patient was taking. The patient was taking torsemide, metolazone, spironolactone, and K supplements at the previously prescribed doses in addition to furosemide 40 mg twice daily. However, per our EHR and the most recent cardiology clinic note (three days before presentation), the patient’s active medications did not include furosemide. The patient quit alcohol use 6 months before the presentation.

On physical examination, the temperature was 36.6℃, pulse was 70 beats per minute and regular, respiratory rate was 18 breaths per minute, and blood pressure was 108/79 mmHg. The O2 saturation was 99% breathing room air. His oral mucosa was dry. The jugular venous pressure was less than 5 cm. The lungs were clear to auscultation, and auscultation of the heart showed a regular pulse, with an S3 gallop and no murmurs. On abdominal exam, bowel sounds were present. There was no abdominal distention and no suprapubic tenderness. The extremities were warm, with 2+ dorsalis pedis and posterior tibial arterial pulses in the feet bilaterally and no peripheral edema.

Lab results were significant for serum potassium of 2.3 mg/dL, sodium of 131 mmol/L, serum carbon dioxide of 38 mmol/L, serum creatinine of 5.0 mg/dL (baseline 1.45 mg/dL), and magnesium 3.3 mg/dL. High sensitivity troponin I levels were elevated at 135 ng/L, repeated at 163 ng/L.

**Figure 1:**
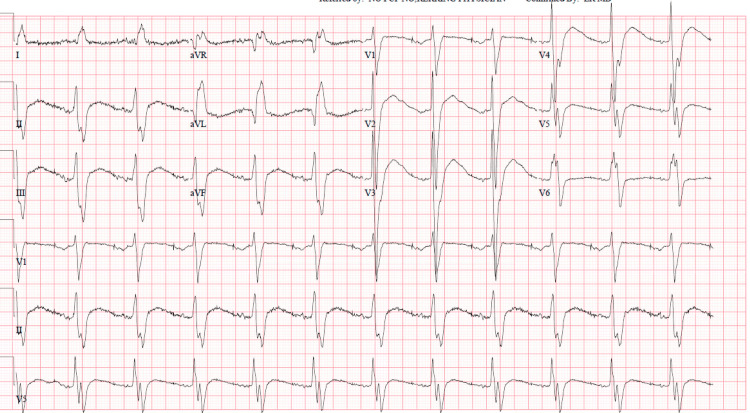
Electrocardiogram showing an atrial-paced rhythm with slowed AV conduction AV: atrioventricular

Electrocardiogram (EKG) showed an atrial-paced rhythm with slowed atrioventricular (AV) conduction (Figure 1). 

**Figure 2:**
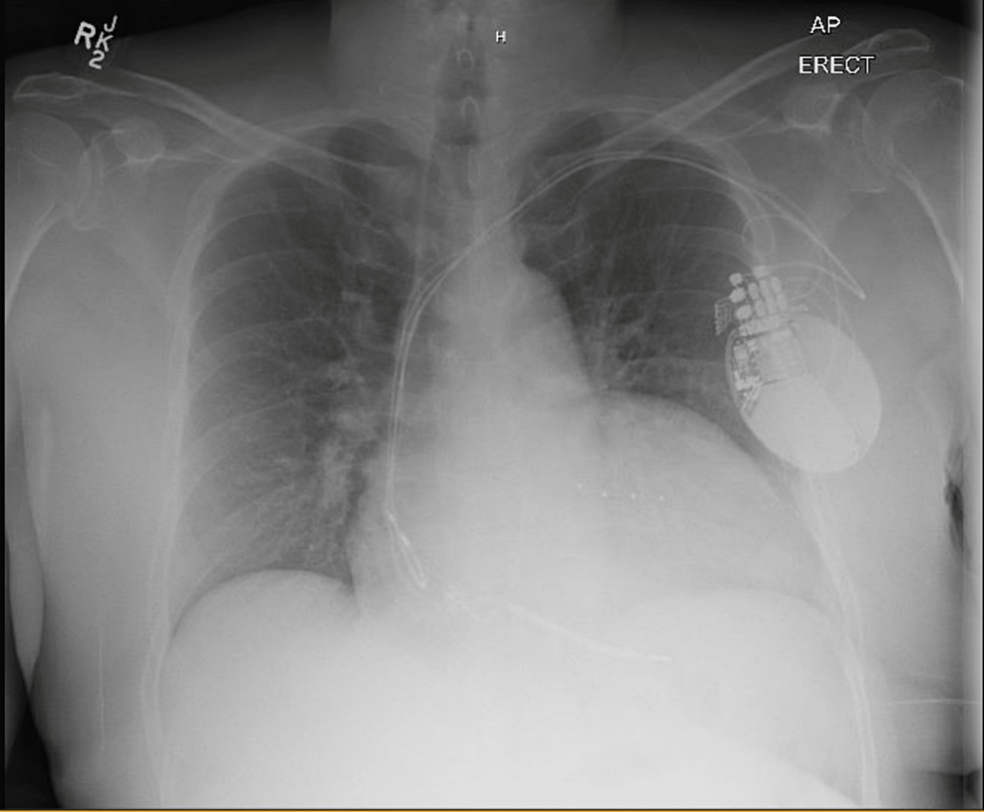
Chest radiograph showing an enlarged heart and an automated intracardiac defibrillator. There is no focal consolidation, pulmonary vascular congestion, pleural effusion, or pneumothorax

Chest radiograph revealed a mildly enlarged heart and no pulmonary vascular congestion. There was no focal consolidation, pleural effusion,or pneumothorax, and the AICD/pacemaker was in place (Figure 2).

Transthoracic echocardiography (TTE) showed severely increased left ventricular cavity size, normal left ventricular thickness, severely decreased left ventricular systolic function, visually estimated left ventricular ejection fraction of approximately 10%, and global hypokinesis which was not changed from his previous TTE (Video 1).

**Video 1:** Transthoracic echocardiogram, apical 2 chamber view

The patient was admitted to the general internal medicine service of our hospital for evaluation and management of his chest pain.

## Discussion

The diagnostic framework for chest pain (or chest discomfort) is broad and can be secondary to cardiovascular, pulmonary, pleural, or musculoskeletal disease; esophageal or other gastrointestinal (GI) disorders; herpes zoster; cocaine use; or anxiety states. Our leading hypothesis was electric shock from his AICD secondary to severe hypokalemia from over-diuresis. We also needed to rule out life-threatening causes of chest pain, such as acute coronary syndrome (ACS), pulmonary embolism, and pneumonia. Acute myocardial ischemia was unlikely; the chest pain was atypical, and although high-sensitivity troponins were elevated, the trend was not significant. The EKG was not suggestive of acute ischemia, and the TTE did not show regional wall motion abnormalities. Pulmonary embolism was unlikely given the presentation of atypical chest pain, appropriate anticoagulant therapy, and a low Wells score (calculated as 0 points). Pneumonia also was unlikely given the absence of fever, cough, chest radiographic findings, and the absence of leukocytosis. We requested an interrogation of his AICD which revealed recurrent episodes of ventricular fibrillation that were successfully detected and shocked by the AICD.

Ventricular fibrillation (VF) is one of the causes of sudden cardiac death in patients with HFrEF. Other important causes include coronary ischemic events and mechanical failure. Fortunately, in patients with HFrEF, sudden cardiac death secondary to VF can be prevented electrically by an AICD [7]. This was clearly demonstrated in our patient who suffered multiple episodes of VF that were successfully defibrillated by the AICD.

Ventricular tachyarrhythmias (VA) are common in patients with HFrEF and the incidence increases with the worsening of the New York Heart Association (NYHA) class [8]. There are different pathophysiologic mechanisms that work together to increase arrhythmogenicity in CHF. These mechanisms may be intrinsic to the heart or extracardiac.

**Figure 3:**
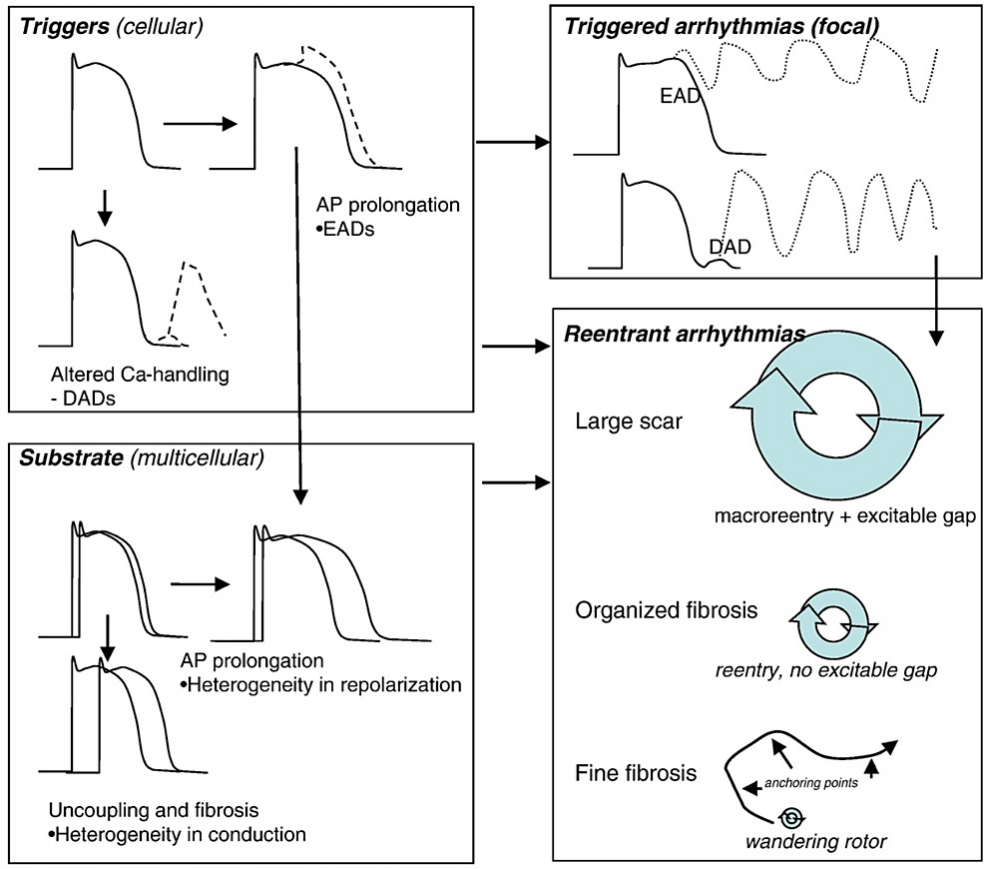
A diagram demonstrating the interaction between the cellular and multicellular arrhythmogenic mechanisms resulting in arrhythmogenesis in heart failure Adapted from Alvarez et al. [9]. In the public domain. AP: Action Potential; EAD: Early After Depolarizations; DAD: Delayed After Depolarizations

Important pathophysiologic processes in CHF that contribute to VA include (1) myocardial hypertrophy, fibrosis, and ischemia; (2) the electrophysiologic changes in CHF including abnormal calcium homeostasis, repolarization, and gap junction remodeling; (3) the changes in neurohormonal signaling and the role of endothelin [9] (Figure 3). Other factors that contribute to VA in CHF patients include severe electrolyte derangements, e.g., hypokalemia, medication side effects, and substance use disorders, e.g., cocaine.

Hypokalemia is prevalent in CHF [4]. It may be attributed to the underlying cardiac disease, pharmacological intervention to manage heart failure, especially diuretics, and associated illnesses [10]. There is data to suggest that hypokalemia affects the cardiac electrical stability in multiple mechanisms which include 1) direct pro-arrhythmogenic effects on ventricular muscle cells, 2) potentiation of medication-induced arrhythmias, for instance, digitalis-induced cardiac toxicity, pro-arrhythmic effects of sympathomimetics, QT prolongation with other QT-prolonging agents (e.g., fluoroquinolones antibiotics), and 3) potentiation of cardiac arrhythmia in the setting of structural cardiac abnormalities (e.g., ischemic heart disease and left ventricular dilatation) [11]. In our patient, diuretic-induced hypokalemia in the setting of structural heart disease triggered recurrent episodes of ventricular arrhythmia (ventricular fibrillation).

We held the patient’s home diuretics and sacubitril-valsartan because of acute kidney injury. We repleted his volume status with cautious intravenous fluid administration and replaced his potassium initially with intravenous and then oral potassium. Fortunately, the patient showed rapid improvement in electrolyte and kidney function. Once stabilized on oral potassium, we discharged the patient on hospital day 6 with plans to follow up (within 2-3 days) with his cardiologist for re-initiation of heart failure goal-directed medical therapy.

## Conclusions

Meticulous medication reconciliation is an important step in avoiding severe hypokalemia and its fatal cardiovascular complications. Both severe hypokalemia and its potential to cause life-threatening ventricular arrhythmias are more common in patients with CHF. AICD is an important preventive measure against sudden cardiac death from ventricular arrhythmia in patients with CHF.
